# Supplementary material for: Using deep learning to predict outcomes of legal appeals better than human experts: A study with data from Brazilian federal courts
Source: PLoS One. 2022 Jul 28;17(7):e0272287. doi: 10.1371/journal.pone.0272287 (PMC9333285; doi:10.1371/journal.pone.0272287)
Supplement: S1 Appendix — (DOCX) [file pone.0272287.s001.docx]

**Datasheet for the Brazilian Courts Appeals Dataset for the 5th Regional Federal Court — BrCAD-5**

**Datasets and pretrained models**: <https://www.kaggle.com/eliasjacob/brcad5> - DOI: 10.34740/kaggle/dsv/3310186

**GitHub repository with code**: <https://github.com/eliasjacob/paper_brcad5>

This datasheet is based on the work of Gebru et al. [1] and answers several questions regarding the creation of the dataset.

**Motivation**

The dataset was created for academic purposes, specifically, to design a set of AI models capable of predicting the outcomes of appeals in Brazilian Federal Small Claims Courts (FSCC) within the 5th Regional Federal Court (TRF5) jurisdiction (DOI: 10.1371/journal.pone.0272287). It was created by Drs. Elias Jacob de Menezes-Neto and Marco Bruno Miranda Clementino exclusively for this research.

The study that created this dataset was funded by the Brazilian National Council for Scientific and Technological Development (CNPq) through a scholarship to Elias Jacob de Menezes-Neto (302668/2020-9). However, the funder had no role in the study design, data collection, analysis, or decision to publish or prepare the manuscript.

The dataset comprises 765,602 appeals tried by Federal Small Claims Courts within the 5th Regional Federal Court jurisdiction in Brazil. A unique case number identifies each case. Each line in the dataset corresponds to an appeal tried between January 2006 and April 2020. We have selected all cases in which the losing party decided to appeal the decision by the first instance federal judge. We applied some preprocessing techniques described later in this document. We present the distribution of labels in the table below:

| **Class** | **Number of events** |
| --- | --- |
| Reverse | 111,276 (full reverse) + 49,259 (partial reverse) = 160,535 |
| Affirm | 605,067 |
| Total | 765,602 |

The dataset is self-contained and includes data gathered by scraping public data from CRETA, an electronic filing system used by all FSCCs within the TRF5. Its jurisdiction encompasses 127 federal courts in six Brazilian states: Alagoas, Ceará, Rio Grande do Norte, Pernambuco, Paraíba, and Sergipe. CRETA automatically excludes from public records all instances where a federal judge decided to seal the case.

According to the Brazilian constitution (article 5, LX, and article 93, IX), court records are considered public and must be publicly available for inspection by anyone. Only in exceptional cases can records be sealed due to their nature or by a judge's decision. Thus, FSCC lawsuits are almost always public, being easily accessible by anyone with an internet connection. While full access depends on providing user credentials, we have used only basic case information, which is freely available. This includes plaintiffs' and defendants' names, case details, and judicial opinions.

Each instance consists of structured and unstructured data, including case metadata and the full text of the decision from both the first instance court and the appellate panel. The column entitled "label" indicates whether the appellate panel affirmed the first instance court decision or reversed it.

In all cases, a plaintiff is a natural person, and the defendant is the Brazilian Federal Government or one of its entities (agencies, foundations, or companies). Unfortunately, there is no information about who is the appellant because the CRETA system does not provide such data.

We have removed the column that corresponds to the plaintiff's name, even though it is publicly available. Thus, one can quickly identify plaintiff names by consulting the CRETA system.

We proposed a time-sensitive split between training, validation, and test dataset. We split the data into subsets after ordering it chronologically based on the date of the decision by the appellate panel. The first 80% of entries were considered training data. We randomly split the last 20% between validation and test.

**Collection process**

We created web scraping software to access every single case from all FSCCs within the TRF5. Our web scraper queried the public record for every case number to gather all publicly available information regarding that case. Dr. Elias Jacob de Menezes-Neto was solely responsible for the data collection process, which took place between December 2019 and April 2020.

Because the judiciary made all case data publicly available, we have not conducted any further assessment other than checking data integrity by randomly evaluating whether or not the scraped data corresponds to the information presented in the electronic filing system.

**Preprocessing/cleaning/labeling**

We had to collect our target variable, that is, the outcome of appeals, from several data points in CRETA. This information may be present within the case metadata, but it was not always available, and, after some manual checking, we discovered that several labels were obviously wrong. Such errors happen when court clerks mishandle cases within the electronic system or when judges make some last minute change to their decision and they fail to rename the file uploaded to the system. We used several heuristics to identify and assess the quality of our labels from three data points: 1) case metadata; 2) filename containing the AP ruling; 3) hand-crafted regular expressions to extract the label from text of the AP ruling. In the end, we used a simple voting classifier in which at least two out of three label sources must agree in order to consider that label valid. We discarded from this dataset all other instances that did not match this criterion.

**Uses**

We used the dataset exclusively for academic purposes to train several machine learning models that can predict the outcome of an appeal based on the full text of the first instance court decision. The code for the paper can be accessed at <https://github.com/eliasjacob/paper_brcad5> and the dataset is available at <https://www.kaggle.com/eliasjacob/brcad5> (DOI: 10.34740/kaggle/dsv/3310186).

**Distribution**

We will distribute this dataset with the academic paper for which it was originally created. We provide the dataset with the Creative Commons Attribution-NonCommercial-ShareAlike 4.0 International Public License. Full details can be accessed at https://creativecommons.org/licenses/by-nc-sa/4.0/

**Maintenance**

Dr. Elias Jacob de Menezes-Neto will host the dataset, and all inquiries related to it should be addressed to **elias.jacob at ufrn.br**. One can check any revisions or erratum at the GitHub repository of the original paper <https://github.com/eliasjacob/paper_brcad5> . Authors do not intend to publish revisions or updates other than in extreme situations or when required by law.

**References**

1. Gebru T, Morgenstern J, Vecchione B, Vaughan JW, Wallach H, Daumé H, et al. Datasheets for Datasets. arXiv. 2018. Available: http://arxiv.org/abs/1803.09010
